# Voices across difference: a qualitative exploration of health-promoting dialogues between older migrant women and researchers in Norway

**DOI:** 10.1186/s12939-025-02618-9

**Published:** 2025-10-08

**Authors:** Prabhjot Kour, Gemma K. Agata, Esperanza Diaz

**Affiliations:** Pandemic Centre, Department of Global Public Health and Primary Care, University of Bergen, Bergen, Norway

**Keywords:** Health-promoting dialogues, Health promotion, Older migrant women, Researchers, COVID-19, Norway

## Abstract

**Background:**

The COVID-19 pandemic underscored the urgent need for more equitable and inclusive health research, particularly focusing on groups underrepresented in public health development, such as older migrant women. Despite increasing population diversity in high-income countries, little research focuses on the health needs and challenges of this group in an inclusive manner. To address this gap, older migrant women and researchers in a Norwegian city participated in sustained health-promoting dialogues over one year. These dialogues aimed to enhance health knowledge among the women and to increase researchers’ awareness of the women’s lived experiences and challenges. Guided by the concept of health-promoting dialogues and health promotion, this qualitative study explored how both groups experienced this dialogue process.

**Methods:**

Data were collected through individual in-depth interviews with eight older migrant women and seven researchers and analyzed thematically.

**Results:**

The analysis identified three main themes: knowledge gain by both groups, dynamics of collaboration, and significance of power dynamics, with related subthemes. Findings show that health-promoting dialogues were experienced as mutually enriching, enabling both groups to acquire new knowledge. Older migrant women developed a deeper understanding of their health conditions and their vital role in contributing to the research while researchers gained more insight into the women´s challenges and strengths. However, persistent language barriers shaped the dynamics of communication in complex ways for both groups.

**Conclusion:**

The study highlights that health-promoting dialogues between researchers and marginalized populations fosters collaboration, mutual learning, trust, and empowerment, highlighting the potential of facilitating such dialogues as a framework for inclusive and health promotion research.

**Supplementary Information:**

The online version contains supplementary material available at 10.1186/s12939-025-02618-9.

## Introduction

The COVID-19 pandemic highlighted how deeply health inequities shape healthcare outcomes during crises [1]. The vulnerabilities of certain groups were magnified, particularly the older adults, many of whom faced profound challenges as they navigated health systems and societal disruptions. Older adults were not only more vulnerable to the virus itself but also to the social and emotional effects of prolonged isolation, disrupted routines, and difficulties accessing healthcare services [2]. At the same time, older adults faced age-based discrimination during the COVID-19 pandemic, experiencing high rates of infection, a decline in self-worth, and being perceived as a burden on health and social services, leading to greater negative impacts compared to younger people [3].

For older migrants, these challenges were even more pronounced. Language barriers, cultural differences, unfamiliarity with health systems, and digitalization of the public services in a bid to curb the spread of COVID-19, added layers of complexity to their pandemic experiences. Health services used digital platforms for communication regarding testing, appointments and vaccination. Because of low digital literacy and additional language barriers, many older migrants relied on family members to log into these health services platforms for tasks that might seem easy to most, as booking a doctor’s appointment [4]. Relatively younger migrants were more susceptible to infection via their jobs, such as care giving jobs, taxi drivers and other service jobs [5], with no option to work from home, further increasing the risk of infection to the older migrants in their households and families [5, 6]. Further, older migrant women were generally more severely affected by the negative consequences of the pandemic [7], facing challenges in maintaining active social life in the host country due to language barriers [8].

Despite increasing calls for more equitable and inclusive health promotion strategies, older migrant women are seldom meaningfully engaged in health research or shaping health interventions that directly impact them. This marginalization limits opportunities for shared understanding and systemic improvement [9]. Yet, their lived experiences and contextual knowledge are critical to understanding the complex intersections of gender, age, migration, and public health in multicultural societies [10].

Health promotion, as conceptualized by the World Health Organization [11], extends beyond providing individuals with medical information or behaviour change tools. Rather, it also involves empowering individuals and communities to gain greater control over the social, cultural, linguistic, and structural determinants of their health in order to reduce health inequities [11–13]. In this regard, engaging older migrant women in dialogue about their lives, needs, and barriers becomes an inherently health-promoting act [14, 15]. Such dialogues affirm the validity of their experiences, generate new knowledge to inform systems-level improvements, and foster relational trust between communities and healthcare professionals [14, 15]. This relational trust is crucial for building participatory and equitable health systems that address the needs of diverse populations, particularly those that have been historically marginalized, underscoring health promotion as a key strategy for advancing health equity [13, 16].

Health-promoting dialogues, therefore, can serve as both a means and an outcome of health promotion [15]. Rather than simply delivering health information, these dialogues are interactive and relational, emphasizing empowerment, mutual understanding, and shared knowledge. They create space for participants to express their own goals, values, and lived experiences, and have been increasingly recognised as a vital component in health promotion strategies. Previous studies have highlighted their effectiveness in various settings [14, 15, 17]. For instance, a Norwegian study among older adults receiving home care highlighted how health-promoting dialogues encouraged a shift from disease-centered approaches to person-centered conversations that reinforced individual strengths and autonomy [13]. Similarly, in Sweden, older adults with multimorbidity felt empowered and acknowledged when engaged in such dialogues that prioritized their emotional well-being alongside physical care needs [16].

Beyond their empowering effects for participants, health-promoting dialogues also function as collaborative platforms for shared understanding and mutual learning. These exchanges help healthcare professionals and researchers to better understand the social, cultural, and linguistic contexts that shape people’s health experiences. Open-ended conversations can allow participants to share insights that improve health professionals’ understanding of structural barriers and community-based solutions [14, 15]. This not only enhances the relevance and quality of health interventions but also strengthens trust, improves service design, and contributes to more inclusive research and policy outcomes, while empowering communities to take ownership and make changes by themselves [18, 19].

When health promotion efforts are examined through an equity lens, it is often seen that strategies that improve population health overall do not necessarily benefit vulnerable or disadvantaged groups to the same extent [13]. This underscores the importance of tailored, inclusive approaches such as health-promoting dialogues that centre lived experience and relational knowledge. Despite their potential, structured health-promoting dialogues with older migrant women remain rare, and existing research on migrants tends to either overlook gendered experiences or conflate men and women into homogenous categories [20]. Further, those that address women issues tend to focus on women of childbearing age, leaving a gap in understanding the unique needs and strengths of older migrant women.

While equity in healthcare is often framed as a policy objective, this study positions equity in healthcare as an emergent outcome of relational and empowering health-promoting dialogues, which foster reciprocal relationships, validate diverse forms of knowledge, and promote individual empowerment through participatory and responsive health systems.

This study, therefore, aims to explore the experiences of health-promoting dialogues between researchers and older migrant women in Norway. We hypothesize that health-promoting dialogues between researchers and older migrant women, as both a means and an outcome of health promotion, providing an opportunity to bridge gaps between marginalized populations and the health systems designed to serve them.

### Older migrants in Norway

According to Statistics Norway, migrants are persons born abroad of two foreign-born parents and four foreign-born grandparents and they contribute to 16.8% of Norwegian population [21]. More than 11% of migrants in Norway are 60 years or older, making up 7% of the country’s total senior population. These older migrants represent 197 different countries [22]. Older migrants are more likely to live in larger households of five or more people, with 7% living in overcrowded housing compared to just 1% of Norwegian older residents. Among older migrants, women outnumber men due to their longer life expectancy, especially in groups from countries like Russia, the Philippines, and Thailand, where over 80% of older migrants are women, primarily arriving through family reunification. However, some nationalities, such as Poland and Pakistan, still have more older migrant men, largely due to labor migration [22]. Further, Statistics Norway’s survey on living conditions indicate that older migrants tend to report poorer physical and mental health than the general population, despite the limited knowledge about many migrant groups in this age range [22].

Norway provides universal healthcare coverage funded through taxation, and migrants who are legally residing in the country are entitled to access healthcare services, including assignment to a general practitioner. The general practitioner serves as the main point of access to most other health services. Interpreter services are available and free of charge for patients with limited Norwegian proficiency, as mandated by the Interpreting Act [23].

It is projected that by 2050, the number of older migrants in Norway will increase tenfold [24]. As the number of older adult migrants continues to rise, healthcare services and staff are expected to see more migrant patients. However, older migrants in Norway currently make less use of both primary and specialist healthcare compared to their non-migrant peers of the same age. They visit general practitioners less often, receive fewer home healthcare services, and have lower rates of using somatic specialist health services and hospital admissions [22]. Moreover, older migrants in Norway report poorer experiences with general practice compared to the majority population, particularly in areas such as communication, accessibility, and coordination of care [25, 26]. These disparities are not solely attributable to individual factors such as language barriers, limited health literacy, or unfamiliarity with healthcare procedures. Rather, they reflect broader systemic challenges, including inconsistent access to interpreter services due to economic constraints, limited cultural competence among healthcare providers, experiences of discrimination, and insufficient targeted outreach to migrant communities [23, 25, 27]. Together, these factors contribute to an environment where older migrants feel less confident navigating the system and may hesitate to seek care, underscoring the need for more inclusive and culturally responsive healthcare practices [23].

## Methods

The study used a qualitative research design to explore the experiences of health-promoting dialogues in a series of regular meetings between researchers and older migrant women in Norway, guided by the theoretical framework of health promotion and health-promoting dialogues.

This study is part of a larger project titled “*Older women with migrant background in Norway: What are their needs in the post-pandemic era?”*, initiated in 2023 by a research centre at a national university in collaboration with a local hospital, a regional university, a nonprofit organization, and the local municipality. For the larger project, female researchers from these institutions gathered once a month over the course of one year with older migrant women with the purpose of learning which health challenges were relevant for them and giving tailored health information about topics chosen by the women, and to better understand how the women viewed the role of researchers and their own position in research. The cut off age for the older migrant women was set at 50 years, even though older individuals are typically defined as those aged 60 and above. This decision was consistent with practices in the literature involving migrants, where lower life expectancy in many of their countries of origin often justifies a lower age threshold [28]. After being explained the purpose of the project, a group of older migrant women in a Norwegian city engaged in monthly health-promoting dialogues with the researchers, getting the possibility of addressing various medical issues raised by the participants. The topics chosen included cardiac diseases, fatigue, healthy aging, musculoskeletal issues, dental health, vaccines, sleep, and mental health, which were discussed across five meetings in the first half of the year. The venue for the meetings was facilitated by the nonprofit organization, as a place that was experienced as known and safe for migrant women. A simple meal was always arranged at the beginning of the meeting. Given the diverse backgrounds of the women, the meetings were conducted in Norwegian. However, ad hoc translations were often provided when needed, either by women with similar backgrounds or by some of the researchers who also had migrant backgrounds. Researchers aimed to present the health topics with an easy language and interactively, fostering dialogue through questions and answers between the migrant women and researchers. For certain themes, researchers or care providers not directly involved in the project were engaged, but only after the project’s intentions were clearly explained to them. The second and third authors were present at all these meetings.

For the purpose of this study, a criterion-based purposive sampling method was used to recruit within the two groups of participants: older migrant women and researchers, for individual interviews. The inclusion criteria for migrant women were to be aged 50 and above -which was an inclusion criterion for the project-, to speak and understand basic Norwegian as defined by the woman herself, and to have attended at least two of the aforementioned meetings. Researchers included in this study were those who presented at the meetings and participated in at least one complete session. The third author of this paper was excluded despite her presenting themes and attending all the meetings.

The second author attended these meetings as an observer but did not present any theme. She informed both the migrant women and researchers about this part of the study within the larger project and invited them to participate in the interviews. This recruitment strategy allowed for direct communication, fostering trust and engagement with potential participants, and facilitated the recruitment of both groups for the interviews.

### Participants

The study included a total of 15 participants, out of which 8 were migrant women and 7 were researchers.

The migrant women were aged between 50 and 74 years and had lived in Norway for over 10 years. All of them had received basic formal education and had professional jobs, such as accountant, nurse, midwife, and dermatologist assistant, in their country of origin prior to relocation to Norway. However, very few of them had managed to work in such positions in Norway. The women originated from the Middle East, South America, Africa and Southern Europe.

The researchers were drawn from different health related disciplines such as psychology, cardiology, dentistry and physical therapy, with 3 out 7 being of migrant background and rest Norwegian born. The experience in their fields of expertise ranged from 9 years to 40 years, with most of them having had prior contact with migrants in their professional circles as well as outside of work.

### Collecting data

Data was collected through in-depth individual and dyadic interviews. The interviews were conducted by the second author between June 2023 to October 2023, in a location suggested by each participant, in order facilitate open and honest communication. The interviews, 9 individual and 3 dyadic, were carried out using semi-structured interview guides.

The interview guides, one for the researchers (Appendix A) and the other for the women (Appendix B) were created by second and third author, consisted of open-ended questions to explore the experience of health-promoting dialogues between older migrant women and researchers.

All the interviews were conducted in Norwegian, audio recorded and transcribed to English. The interviews lasted between 45 and 60 min and were conducted in locations chosen by the participants, including our office space, their homes and local NGO’s office.

To ensure privacy, we followed the participants’ preferences and selected settings that allowed for confidential and uninterrupted conversations. In all cases, we ensured that the interviews were conducted in environments where participants felt safe and comfortable sharing their experiences. Additionally, we reminded participants of their right to pause or stop the interview at any time, and they could withdraw from the interview.

The interview transcripts were then anonymized and securely stored on a protected server, SAFE. The participants were assigned numbers (W for migrant women and R for researchers), which were used in the presentation of results.

### Thematic analysis

Following the aim of the study, the transcribed data from interviews was analysed using thematic analysis [29]. The main goal of the analysis was to provide new insights by presenting the participants’ experiences as they expressed them, without probing for deeper, underlying meanings. The emphasis was purely on the semantic level. The thematic analysis was done using the following steps: firstly, the transcripts were thoroughly read several times to become familiar with the data by first and second author. In second step, the data was systematically coded throughout the entire dataset, creating initial codes and gathering data associated with each code. After coding and organizing the data, the codes were grouped into potential themes, which were then reviewed again by the all the authors. Following this, the entire dataset was read once more independently by the first and second author, and the themes were refined in alignment with the study’s objectives. Finally, a comprehensive review was conducted for each theme by systematically arranging relevant data extracts along with corresponding quotes by all the authors, which formed the results section of this paper.

### Reflexivity

All authors of this study are migrants living in Norway and bring lived experiences of migration to their research practice. This shared background has informed both the design and interpretation of the study, fostering a deeper understanding of the complexities surrounding migrant health. The first and last authors have medical background and extensive experience working on projects related to migrant health, integration, and access to healthcare services. The second author has a background in social anthropology. The data collection and analysis were conducted with an awareness of the potential power dynamics between researchers and participants. While all authors share lived experiences of migration, they do not belong to the same ethnic or cultural communities as the participants. This positionality was acknowledged throughout the research process, and the team engaged in reflexive discussions to remain attentive to their own experiences, assumptions, and professional roles that may have influenced the research process and engagement with the participants and the data.

## Results

**Fig. 1:**
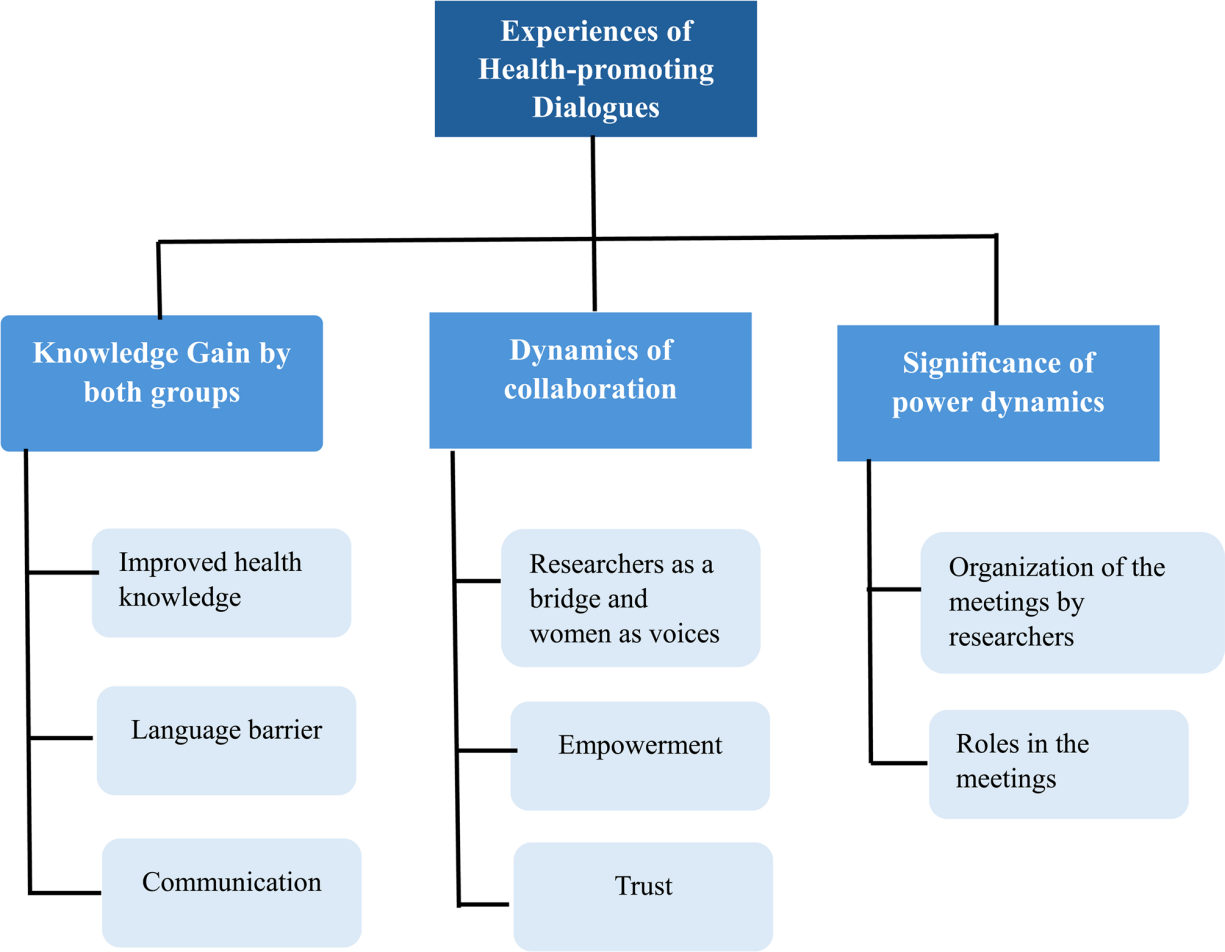
Themes & associated sub-themes

The findings generated in the form of three main themes and associated sub-themes (Fig. 1), emphasized the experiences of health-promoting dialogues for both the older migrant women and the researchers. The main themes were (1) *Knowledge gain by both groups*, (2) *Dynamics of* c*ollaboration*, and (3) *Significance of power dynamics*.

1. Knowledge gain by both groups

Both groups of participants shared that they gained useful knowledge about health and the healthcare system through the health-promoting dialogues that took place during the meetings. While some participants noted difficulties in understanding the language used, others emphasized the importance of adapting one’s way of speaking to ensure the message was effectively communicated. Researchers, in turn, began to reflect on their own role in health promotion, gaining deeper awareness of how language, cultural references, and community-based knowledge influence health behaviours and perceptions. These mutual reflections highlighted the dialogical nature of the meetings, where learning flowed in both directions.

- Improved health knowledge: “I learned a lot”

When describing their experiences at the meetings, most of the migrant women expressed positive sentiments, and some even called the meetings ‘a course’ where they were learning.

*It was very good and nice information*,* for me it was the first time to hear about this information.* W2

The women acknowledged receiving new knowledge on health problems, mainly from the presentations given by the researchers, on agreed topics, and from each other as they answered questions. This knowledge was not only on ailments, their signs and symptoms, how to manage or prevent them, but also on the way the Norwegian health system works.

..*so*,* I learnt a lot about one’s health and explanation of how you experience* (signs and symptoms) *because sometimes I do not understand very much about health and such things* (the health system) W4

Some of the women mentioned that learning new medical words in Norwegian was a positive experience, as it not only increased their knowledge in health but also improved their proficiency in Norwegian, which is not their first language.

*It was a chance to speak more Norwegian and get some special words* (medical terms) *that are not used in daily life.* W1

b) Language barrier: “my friend helped me”

The meetings were conducted in Norwegian, which was the second or third language for the migrant women and as such there were challenges in understanding, as described by the women. They reported challenges with some words used in the presentations, since they were not everyday words that they could easily relate to. However, they could ask for explanations and once these were translated, mostly by their peers and sometimes by other researchers present, they had no problem in comprehending the presentations.

*for me it was hard why*,* so they speak bit by bit (simplified explanations)*,* but it is hard because I do not know how to speak well*,* my friend helped me…the language for me was difficult not that what they were saying.* W2

The complexity of Norwegian language was a concern to some of the women. Like most languages, Norwegian has several dialects with different nuances and for some women this was a challenge. The different pronunciations made it difficult to understand as one participant stated.

*Not the language exactly*,* because I think it’s a dialect*,* there are different people speaking different dialects*,* you see*,* but foreigners (migrants) speak Norwegian and I understand*,* you see. You understand.* W5

The need to be understood when interacting with healthcare personnel out of the meetings was brought up by a participant who described her encounter with her general practitioner. She mentioned similar words used at the meetings as being also used when interacting with healthcare personnel and expressed this as being of immense help.

*Sometimes I have a conflict with the GP because they don’t understand foreigners … the Norwegian GP doesn’t have good communication with me*,* I use an interpreter*,* but the interpreter cannot explain what I feel… but you (researchers) explained a little some topic that helps many foreign women because they (GPs) sometimes use words like those used at the meetings.* W5

For the researchers, concerns related to language were two-fold, first that it was not the first language of the migrant women and second having to simplify technical language to non-technical language. When describing their thoughts on how they disseminated information during the meetings, some researchers acknowledged the challenge of breaking down professional jargon to simple language especially to an audience to whom it is not the first language.

*I think mostly because it is easy to forget how difficult it is to understand another language … and I think the scientific language is one thing*,* but I think the…. when you don’t have Norwegian as a main language then it is double difficulty.* R4

Some researchers also reported being uncertain as to whether the information they gave was understood as they meant it to be understood because of the language issues.

*I do think that the… language made it probably a bit more difficult because I wasn’t quite sure if they received the way I tried to explain it*,* so of course that is (I am) always thinking when there’s different languages that’s always a possibility that I haven’t been able to sort of convey what I wanted to convey and that they may misunderstand what I was saying.* R7

c) Communication: “I would have completely missed their way of speaking”.

Although for some researchers communicating with the migrant women was not difficult, others reported challenges with the intricacies of communication. A researcher reported learning how to communicate with this particular group after interacting with them in several meetings before making her own presentation, because communication is more than just giving information.

*I was very happy for having been in the room before because if not I think I would have completely missed their (migrant women) way of speaking mostly because I think it is easy to forget how difficult it is to understand another language.* R4

Some participants mentioned that just by being present at the meetings and interacting with each other (researchers and women) opened channels of communication for them that would otherwise have not been possible if they had not had this opportunity.

*I certainly met people I would not have met if I didn’t participate in this program… while eating at the beginning or at the end of the program and so forth that we discussed normal things*,* you know like hairdressing*,* wigs or like normal things.* R1

2. Dynamics of collaboration

The desire to collaborate and be part of something by giving one’s voice was expressed by the participants, especially the migrant women. Participating in the meetings was perceived by all participants as an opportunity for collaboration and learning from each other.

- Researchers as a bridge and women as voices: “it is a starting point to find a way to present the problem through researchers”

All the migrant women said they would recommend others to participate in such projects as this. One of the main reasons given was to create public awareness of their health concerns by using researchers as a medium to reach the mainstream society.

*I can tell them that when there is a research project and it is about something they know or a problem that they have experienced*,* for example*,* we as migrants*,* you (one) can sit at home with so many worries and do not know who or where to ask for help but if a researcher asks you*,* it is as a starting point to find a way to present the problem through researchers*,* therefore I can recommend becoming a participant.* W8

Participating in these meetings, with researchers, was also seen as a way of collaboration in bringing forth their health concerns as users of the health system, to the health authorities by some of the women.

*I think it was important that we participated in this course(the meetings)*,* it might give you (researchers) something that you can take to those in charge (health authorities) of all the topics that we talked about*,* so if only (you) take our problem to them*,* and this research that you have*,* or projects (it) will help us in the future (and) not just us.* W1

For some researchers participating in the meetings was a reminder of the diversity in society that calls for mutual learning and creating knowledge together between researchers and members of society.

*I hope that we can know*,* and we can know and write papers and that*,* but it is what happens in a society really that is also a bit of change in the research field*,* so it is*,* I think*,* very… how do you say action way I mean*,* I meant cocreation. I mean it’s a buzzword but actually it is true that it is cocreation of knowledge.* R4

b) Empowerment: “I feel confident now I have knowledge”

Some women mentioned their new-found confidence as a result of acquiring knowledge from these collaborative meetings, which were experienced as empowering.

*I feel confident now I have knowledge*,* not before (had no knowledge before the meetings).* W7

For other women, the information they received at the meeting gave them a sense of security in the knowledge that they have some understanding of their own health.

*I feel safer because of the information I got; the topics are important to women over 50 years. I did not know so much (before)*,* it is very important.* W7

Some of the researchers acknowledged the migrant women had some knowledge, that they lacked. One researcher expressed that the knowledge of migrant women can be used as a helpful resource for research.

.*they have knowledge that we need also when we are making new research programs.* R5

The said knowledge also points them in the direction to carrying out relevant research as mentioned by another researcher.

.*that was interesting to me to see what health problems they are concerned about or know of.* R1

c) Trust: “we have found a common thing for us and that is that sharing room”

Trust was important in enabling the sharing of experiences and ideas during the meetings, as reported by both the researchers and the migrant women. The researchers were viewed as a credible source of information by the migrant women, as opposed to the many available sources of information out there, such as the internet or friends.

.*but it’s different (what) you hear on the street you hear a lot*,* right*,* not (like) from that doctor and the good doctor specialist that explain*,* right I never heard from specialist ok*,* this ticked (boxes).* W-7

The need for fostering trust was also important to the researchers and not just for data collection, but for creating a relationship and opening channels for cooperation in future, as explained by one researcher:

..*such projects I think they have two benefits: that you can get data yes*,* and at the same time it will improve the communication between you as a researcher and other participants.* R2

According to the participants, a trusting relationship between them (the researchers and the women) made collaboration much easier as it gave room for open self-expression and mutual understanding.

..*we have in that particular room you have like over seven different backgrounds and then I mean*,* and then we have found a common thing for us and that is that sharing room*,* that sharing room it says it has created a big what would I say yeah “trust” what is “trust”.* R6

The trust built between the participants led to them having a pleasant experience full of respect and good relations with each other during the meetings as expressed by one of the women.

*All of us had a good time*,* we helped each other and had good relations with each other*,* and I experienced (this) for the first time*,* there was a lot of respect for each other in the room. I am very happy.* W5

Having a small meal together created room for the participants to familiarize themselves with each other in a social environment devoid of the differences in professional or cultural backgrounds. This also made opening up much easier for the participants as one of them described.

..*and also*,* that we always started with eating something together you know it’s easy to have the small talk going*,* over a cup of coffee or a small meal you can discuss a little easily.* R1

3. Significance of power dynamics

Power differences were not directly mentioned but implicitly expressed in different ways by the participants.

- Organization of the meetings by researchers: “I needed a little more time”

For some participants, mostly the researchers, the time scheduled for the meetings, and their duration was convenient and enough for them to meet their objectives of giving information to the women, at the same time learning from them.

For the women, the objectives were comprehending the information and getting clarifications on health issues. They, however, felt that the time was not enough and that too much information was delivered in too short time leading to information overload, as expressed by these two participants:

*I needed a little more time for the meetings because there was a lot of information and more time (would have been) much better. W5*

*It wasn’t enough for questions. I needed*,* I think 2 h (would have been) enough for questions* W2

Apart from the duration of the meetings not being enough, the timing of the meetings seemed to be a challenge to some women who would rather have it on a day when they could concentrate better and maybe get more out of it, because they did not have to worry about not fulfilling their other roles as members of a family.

*It was for one hour and late*,* from 5pm to 6pm*,* many had problems going home because of the children and family so they had to rush from work because they have not been at home. If it was during the free time (not at work)*,* like during the day on Saturday. Those presenting also rush from work*,* for an hour it was a short time*,* it would have been good if it was two or three hours. For example*,* those who do not work or are free from work can organize things at home and come and enjoy and meet others and talk without stress.* W8

b) Roles in the meetings: “we still have two roles”

While the researchers had the role of making presentations, and the women joined in during the question-and-answer session and to explain things to each other, some participants expressed the wish to have had an opportunity for the women to have a similar presenter-role so they could hear from them too. Even though all participants got a chance to express themselves in one way or the other, some expressed that some participants did not get to contribute as much.

*I think it was good but*,* I would also like to hear from the participants (the women)*,* what they have*,* for example what their thoughts are…*W3

The sentiment of hearing from the migrant women was also mentioned by one of the researchers who expressed the notion of how interesting it would be to hear from them too. This would be a real exchange of ideas.

*It could be interesting also where the other women that are not from the university and health where they present things because then it will be more*,* we still have two roles so it is a bit of not a hierarchy but it is two different roles so it would be interesting if we had kind of had the same roles.* R4

## Discussion

This study explored the experiences of health-promoting dialogues between older migrant women and researchers, revealing how such dialogues served as a platform for collaboration, mutual learning, trust-building, and empowerment. The novelty of our approach relies on the inclusion of the experiences of both researchers and older women. Our findings pointed to three interrelated themes: knowledge gain by both groups, the dynamics of collaboration, and the significance of power dynamics. These themes collectively illuminate the transformative potential of health-promoting dialogues, not simply as an exchange of information, but as a relational process that fosters shared understanding. For such dialogues to be meaningful, all participants must feel that they bring relevant knowledge or lived experience to the table, contributing to a sense of value and inclusion [30]. At the same time, these dialogues are shaped by underlying power dynamics, raising critical questions on how voices across difference and influence are negotiated within the collaborative process [31].

### Health-promoting dialogues as a space for mutual learning

Our findings show that health-promoting dialogues served as more than just platforms for information dissemination. They became spaces of mutual learning and shared understanding. For some of the older migrant women, participating in these dialogues led to tangible improvements in health-related understanding, language use, and confidence in navigating the health system. Importantly, the sessions revealed a diversity of health literacy levels among participants, challenging the homogenizing narrative that migrants as a group possess uniformly low health literacy [32, 33]. In line with Berens et al. [32], our study highlights these nuances within migrant populations, underscoring the need to avoid collective generalizations in both research and practice. Rather than framing migrants as passive recipients of care, these dialogues made visible how their varied experiences, knowledge, and cultural contexts could actively contribute to health promotion efforts.

Health literacy, as observed in this study, is not merely an individual trait but shaped by the structural and communicative environments within which people interact. Researchers began to recognise their own role in promoting what Farmanova et al. describe as organizational health literacy, the responsibility of systems and professionals to adapt health communication and service delivery to meet the needs of diverse communities [34]. This recognition emerged gradually, facilitated by regular meetings held in trustful, informal settings that encouraged open exchange. As researchers learned from the women’s lived experiences, they gained insight into how language, cultural references, and community-based knowledge influence health behaviours and perceptions, which in turn fostered greater cultural awareness, humility and competence. This exchange could lead to the spirit of co-production, where power and expertise are shared and co-created rather than unilaterally delivered [35].

Language played a central role in shaping participation during the health-promoting dialogues. While some participants supported each other through informal translation, language barriers were amplified by complex terminology, often limiting full engagement for several women, echoing previous research on the link between language proficiency and health outcomes [36, 37]. However, these dialogues helped shift the communication burden from being solely on migrant women as individuals responsible for learning the local language to being a shared responsibility. Researchers began to reflect on their language use and adopt strategies such as simplification, visual aids, and culturally adapted communication styles. The collaborative nature of the meetings was further supported by social dynamics like shared meals, humour, and personal storytelling, which helped build trust and dismantle hierarchical roles. As Kilanowski [38] notes, these relational elements are essential for meaningful intercultural engagement. In this way, such dialogues became more than educational spaces, they fostered inclusive, respectful, and co-owned processes of health promotion.

### Collaboration across difference in health-promoting dialogues

Health-promoting dialogues, offer a powerful model for collaboration across difference, whether in terms of culture, language, age, education, or life experience. In our study, these dialogues became spaces where older migrant women and researchers came together not as experts versus laypeople, but as partners bridging complementary forms of knowledge. Such form of collaboration across difference demands an intentional effort to create inclusive conditions for participation, thereby creating a sense of ownership and commitment, but is also empowering by giving all involved a voice and building trust [39]. For the women, participating in the meetings deepened their understanding of research’s relevance to their own health and that of their communities, motivating them to encourage broader participation. They also recognized their role as active users of the health system, reinforcing the value of their engagement. At the same time, researchers were seen by the women as bridges to wider society, conduits through which their voices could be heard beyond the room. Researchers, in turn, valued the women’s voices as essential forms of knowledge, and were prompted to reflect critically on their assumptions and acknowledge the evolving diversity of society, which calls for more inclusive and collaborative research practices. This is in line with the previous study illustrating that new knowledge emerges and mutual learning takes place when professional expertise and community experience engages [40].

Building trust has been central to facilitating the collaboration across difference [41, 42]. Through consistent interaction, shared meals, informal conversation, and an atmosphere of mutual respect, researchers and older migrant women were able to form relationships grounded in trust rather than hierarchy. As reported in other studies, these humanizing elements helped participants feel safe to share and engage more deeply [41, 42]. Trust was reciprocal, while the women expressed confidence in the researchers’ expertise, the researchers also developed trust in the women’s narratives, recognizing their validity and importance. However, as others have noted, trust is fragile and must be continuously nurtured through reciprocity and presence, especially in these dialogue settings [43].

Trustworthy information is key to empowerment, as informed individuals are more likely to contribute significantly, as demonstrated by the participants in our study. Empowerment, often assumed to flow from professionals to communities [44], but in our study, both groups were empowered through the health-promoting dialogues. The older migrant women gained not only health knowledge but also the confidence to interact more comfortably with the health system. At the same time, researchers acknowledged the valuable experiential knowledge and insights that the women brought, which could shape more inclusive research practices in future, thereby recognizing the importance of cultural awareness and humility. This aligns with Abma and Broerse’s view of empowerment as mutual learning [45]. While the empowerment observed was primarily individual rather than structural [46], the experience highlighted how health-promoting dialogues can function as transformative spaces for collaboration across difference, that is grounded in trust, shared purpose, and mutual recognition.

### Unpacking power dynamics in health-promoting dialogues

While health-promoting dialogues were designed as inclusive and collaborative spaces, our findings underscore that power dynamics remained a persistent undercurrent, shaping how participants experienced and engaged in the process. These dynamics can be influenced by age, education, cultural background and life experiences. Such diversity, while enriching, also introduces incongruities that can be challenging to establishing a balanced collaborative process [35]. The power dynamics were especially visible in our study in the way meetings were organized and in the roles that researchers and older migrant women assumed during the meetings.

The organization of the health-promoting dialogue meetings highlighted a mismatch in priorities, while researchers found the duration adequate for sharing information and learning from participants, many older migrant women felt rushed, overwhelmed by the volume of content, and constrained by competing responsibilities that limited their engagement. These differences suggests that logistical decisions, such as meeting time, length, and day are not neutral but carry implications for participation and power, by unintentionally reinforcing power imbalances in collaborative settings [47]. Ensuring that organizational aspects are co-designed with participants could support more equitable and effective health-promoting dialogical spaces.

Power dynamics inevitably shaped the roles in health-promoting dialogues, determining who spoke, when, and for how long. Both groups in this study agreed on having distinct roles. Despite the migrant women choosing the topics, the researchers, by making presentations, were seen as initiators of the dialogue, thus having the power to guide the conversation. They also held expert power, being more knowledgeable about the topics. This aligns with a Norwegian study which found researchers tend to hold more power due to their roles [31]. Researchers may also be seen to have referential power given to them by society and, by extension, the older migrant women. This form of power arises from being perceived as a respected and trusted member of the community, and while it can contribute to power differences in intercultural dialogues, it should not necessarily be seen as negative. In fact, referential power has been shown to positively influence motivation and participation [48] and was somehow acknowledged by the women who preferred to obtain knowledge “directly from experts”. Recognizing such dynamics is crucial for deepening collaboration across difference. Health-promoting dialogues must be more than a platform for knowledge exchange, it must also function as a mechanism for negotiating power, adjusting structure, and continually reassessing who gets to speak, decide, and lead.

### Strengths and limitations

Our study contributes to addressing a gap in research on the experiences of health-promoting dialogues between older migrant women and researchers in Norway, especially considering the limited number of studies that explore the health-related experiences of older migrant women. A key strength of our research is its focus on interviews with both migrant women and researchers, offering in-depth insights into collaborative process through health-promoting dialogues. The study is also timely, given the growing need for research into adapted health promotion interventions and programs aimed at reducing barriers to healthcare access for marginalized groups and promote equity. By focusing on the participants’ experiences, our study responds to recent calls for more evidence on the effectiveness of these interventions and their impact on users and their families. However, the study’s transferability is limited to similar groups and contexts, as it captures the specific experiences of older migrant women and researchers in Norway. Another limitation is that the interviews were conducted in Norwegian, which was not the first language for either the participants or the author who conducted interviews, possibly leading to a loss of cultural nuances when translating thoughts into Norwegian. Nevertheless, the findings are credible and grounded in the participants’ narratives, offering valuable insights into this understudied group.

## Conclusion

The health-promoting dialogues between older migrant women and researchers proved to be a meaningful space for mutual learning, trust-building, and reflection. For the women, the process enhanced their understanding of health and reinforced their role as active participants in the healthcare system. For the researchers, it offered critical insights into how lived experience, language, and cultural context shape health behaviours and access. While challenges such as language barriers and researcher-led formats persisted, these dialogues underscored the transformative potential of collaborative engagement across difference and value of inclusive, participatory approaches in health promotion.

This study highlights the potential of relational dialogues to elevate the knowledge and voices of marginalized groups like older migrant women while contributing to cultural awareness, humility and competency among researchers. It calls for health promotion strategies that are context-sensitive, ethically grounded, and recognize the lived experiences of those they aim to serve. Future research would benefit from embracing the collaborative principles early on, ensuring that diverse voices help define not only the questions being asked but also the methods used to explore them in health promotion initiatives through equity lens.

## Supplementary Information

Supplementary Material 1.

## Data Availability

The interview guide is included as supplementary material. The datasets generated and/or analysed during the current study are not publicly available due to privacy or ethical restrictions.
