# Supplementary material for: Voices across difference: a qualitative exploration of health-promoting dialogues between older migrant women and researchers in Norway
Source: Int J Equity Health. 2025 Oct 8;24:258. doi: 10.1186/s12939-025-02618-9 (PMC12505572; doi:10.1186/s12939-025-02618-9)
Supplement: Supplementary file 1 — Supplementary Material 1. [file 12939_2025_2618_MOESM1_ESM.docx]

**Title: Voices across difference: A qualitative exploration of health-promoting dialogues between older migrant women and researchers in Norway**

***Interview guide for researchers:***

Name

Field of expertise

Duration of work in the field

Experience with immigrants before meetings

Thematic questions:

1. What do you think of the dialogue with the migrant women?
2. How was it for you to simplify technical language for lay people to understand (Probe- difficult/easy how and why)
3. How do you think the information you disseminated was received?
4. Was there any information that came up during the dialogue that was new to you? (Expound on the info)
5. Do you feel like you got a better understanding of this group (Please explain why/why not and how it is different from before the dialogue).
6. How do you think this experience will contribute to your research in future?
7. Were there any questions asked that were challenging to answer and how did you handle that?
8. Did the organization of the meeting work well?
9. Is there anything else about the experience that you would like to talk about?
10. Do you have any questions for me or anything you would like to add?

***Interview guide for older migrant women:***

Age

Occupation

Country of origin

Years of residency in Norway

Reason for migration

Thematic questions:

1. How many meetings did you attend?
2. Can you describe your experience in the meetings?
3. How would you describe the language used to explain the issues? (Probe was it easy/difficult to follow and describe how so).
4. As much time as possible to talk with the other participants and ask questions was given. How was your experience with this? (Probe to describe or explain instances when you did or did not).
5. Can you give some examples of questions, yours, or the other participants, that were or were not answered adequately?
6. How has your understanding and perception of research changed from before the meetings, if it has changed in any way?
7. How do you feel about participating in this research project?
8. Is there anything else you would like to share about your experiences that we haven’t discussed?
9. Do you have any questions for me or anything you would like to add?
